# Transmission of multidrug-resistant organisms by VA CLC residents: A multisite prospective study

**DOI:** 10.1017/ash.2023.396

**Published:** 2023-09-29

**Authors:** Lona Mody, Kristen Gibson, Marco Cassone, Sanjay Saint, Sarah Krein, Julia Mantey, Mary Janevic, Alexandria Nguyen, Taissa Bej, Oteshia Hicks, Lillian Min, Andrzej Galecki, M Todd Greene, Laxmi Chigurupat, Robin Jump

## Abstract

**Background:** Veterans Health Administration (VHA) community living centers (CLCs) provide postacute and long-term care. CLC veterans visit myriad locations outside their rooms (eg, rehabilitation, dialysis). Pathogen transmission during out-of-room visits is unknown. **Methods:** We recruited newly admitted veterans at 3 CLCs. After obtaining informed consent, we cultured nares, groin, hands, and 7 surfaces in the patient rooms. We accompanied veterans to up to 5 out-of-room visits and cultured patients’ hands and surfaces they touched. We tested for multidrug-resistant organisms (MDROs) including methicillin-resistant *Staphylococcus aureus* (MRSA), vancomycin-resistant *Enterococcus* (VRE), and quinolone, carbapenem, and/or ceftazidime-resistant gram-negative bacteria (R-GNB). We defined transmission as a positive culture following an initial negative culture during the same visit. **Results:** We enrolled 137 veterans (median follow-up, 29 days; mean, 5.9 visits); 97% were postacute patients. We conducted 539 patient-room sampling visits (mean, 3.9 per veteran; 5,490 swabs) and accompanied 97 veterans to 266 out-of-room sampling visits (mean, 2.7 per veteran; 2,360 swabs). Of 137 patients, 47 (35%) were colonized with an MDRO at enrollment and 74 (58%) of 128 patients were colonized on any follow-up patient-room visits. Of 133 patients, 55 (41%) acquired a new MDRO, most often VRE (31 of 97, 32%). In patient rooms, toilet seats [114 (21%) of 538], curtains [101 (19%) of 530] and bedrails [98 (18%) of 539] were most frequently contaminated. Among 266 out-of-room visits, 17% had surfaces contaminated with MDROs, most commonly involving dialysis [4 (31%) of 13], radiology [2 (25%) of 8], and rehabilitation therapy [29 (18%) of 159] (Fig. 1).Transmission of MDROs during out-of-room visits was common and occurred in 18% of visits with 8% (9 MRSA and 12 VRE) acquiring a new MDRO on their hands and 12% (9 MRSA and 23 VRE) of MDRO transmission occurring from hands to a surface that the patient touched (Fig. 1). In 18 (58%) of 31 cases, the organism transmitted to a surface was on patient hands at the start of the visit. Transmission was most common during visits to dialysis (3 to patients and 2 to surfaces), radiology (1 to a patient and 2 to surfaces), and rehabilitation therapy (13 to patients and 21 to surfaces) (Fig. 2). **Conclusions:** New MDRO acquisition during VHA CLC stay was common, and nearly one-fifth of out-of-room visits resulted in MDRO transmission. Our analyses suggest that veterans’ hands may shed MDROs (MRSA and VRE) to surfaces. Interventions to reduce MDRO transmission during visits for rehabilitation, dialysis, and other therapies are needed.

**Figure:**
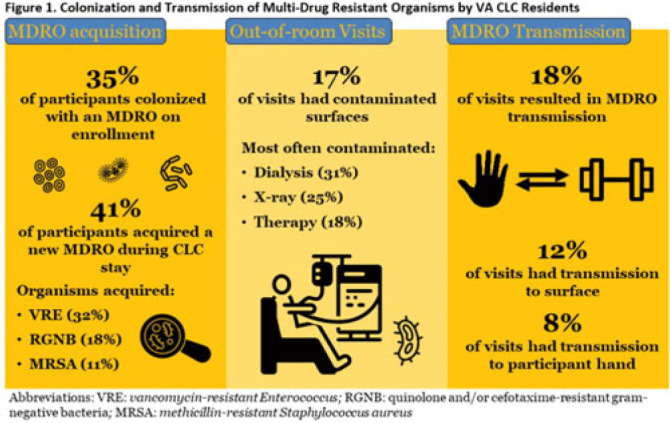

**Figure:**
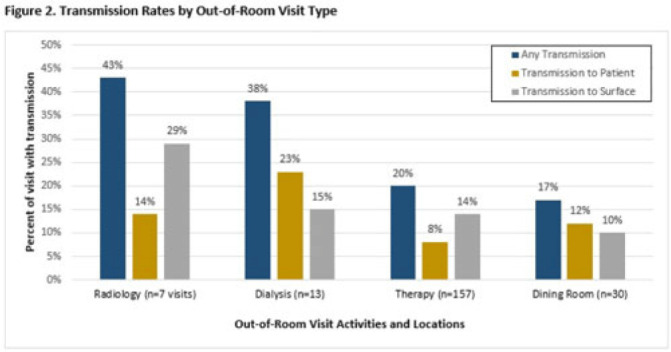

**Disclosures:** None

